# Supplementary material for: Navigational health literacy and health service use among higher education students in Alentejo, Portugal - A cross-sectional study
Source: PLoS One. 2025 May 15;20(5):e0322181. doi: 10.1371/journal.pone.0322181 (PMC12080760; doi:10.1371/journal.pone.0322181)
Supplement: Attachment — Submitted filename: Answers to reviewer 2.docx [file pone.0322181.s002.docx]

Response to Reviewer #2

Dear Reviewer,

We appreciate your constructive feedback and critical insights into our study. Your comments have allowed us to further refine the manuscript, ensuring greater clarity, methodological rigor, and alignment with the study objectives. Below, we address each of your points in detail and outline the revisions made accordingly.

1 – Justification for the Inclusion of the Santarém School

The inclusion of the Santarém school in this study is justified by its classification within NUTS II Alentejo during the 2022/2023 academic year. At that time, national statistical data classified Santarém as part of the Alentejo region. However, we acknowledge that, following the implementation of Commission Delegated Regulation (EU) 2023/674, Santarém has been reclassified under NUTS II Oeste e Vale do Tejo as of 2024 (Compete 2030). Given that our study was conducted in the 2022/2023 academic year, when Santarém was officially designated as part of NUTS II Alentejo, its inclusion remains methodologically sound and consistent with regional statistical classifications at the time of data collection.

2 – Justification for the Sample Size (952 Participants)

The sample size determination was based on standard statistical procedures used in similar studies (Pedro et al., 2023b; Sørensen et al., 2013). Specifically, we applied a 95% confidence level, assumed a population proportion of 50% (to maximize variance and ensure a conservative estimate), and set a 3% margin of error.

After applying the finite population correction, the minimum required sample size was calculated as 952 students. To account for an expected 20% non-response rate, we increased the target sample size to 1143 students. However, due to a higher-than-expected response rate, the final dataset comprised 1979 participants, exceeding the initial requirement. This increase in sample size enhanced the study’s statistical power, improved subgroup representativeness, and allowed for more precise estimates.

Revision in the manuscript: We have expanded the "Sample Size Determination" section to explicitly reference the statistical methodology used and cite relevant literature supporting our approach.

3 – Weaknesses of the HLS16 Compared to HLS47

The HLS-EU-Q16 is a validated short-form version of the HLS-EU-Q47, designed to provide a practical and efficient assessment of health literacy. However, a known limitation of using the 16-item version is that it does not capture the full range of dimensions covered by the original 47-item version. In particular, there is a reduction in the granularity of assessments related to disease prevention and health promotion (Pedro et al., 2023b).

Despite this limitation, prior research (Pedro et al., 2016; Pelikan et al., 2022) has demonstrated that HLS16 remains a valid and reliable instrument for assessing general health literacy levels, making it an appropriate choice for large-scale population studies.

Revision in the manuscript: We have expanded the "Health Literacy Assessment Tools" section to acknowledge the trade-offs associated with using HLS16 instead of HLS47, citing relevant literature (Pedro et al., 2016, 2023b; Pelikan et al., 2022). The HLS16 is also advantageous because it is shorter, i.e. it is easier to answer (making it faster) and another advantage is that more answers can be obtained (participants may not give up halfway through because it is shorter).

4 – Explanation of the Increase from 1143 to 1979 Participants

The increase in final participation numbers (1979 students vs. the estimated 1143) was primarily due to a higher-than-expected response rate. Several factors contributed to this:

- The online data collection method allowed for a broader reach and facilitated participation.
- Institutional support and engagement strategies encouraged student involvement.
- A greater willingness among students to participate in health-related studies may have contributed to the high response rate.

This larger sample size strengthened the study’s statistical power, ensuring greater representativeness and allowing for more nuanced subgroup analyses.

Revision in the manuscript: We have added a note in the "Study Sample" section explaining the discrepancy and its positive implications for data robustness.

5 – Justification for Including a Question on Prior Health-Related Education

Although the study focused on undergraduate students, the inclusion of a question about prior health-related education was based on established evidence indicating that exposure to health-related content can significantly enhance health literacy (Amaral et al., 2021). By including this variable, we aimed to explore whether previous formal training in health sciences influenced health literacy levels, thereby allowing for a more comprehensive analysis of potential determinants.

6 – Future Research on Students with Unsatisfactory Health Perceptions

We agree that the high proportion of students who perceive their health as unsatisfactory warrants further analysis. Disaggregating the data will allow for a more detailed understanding of the physical, mental and social dimensions of self-perceived health, making it possible to identify specific determinants that influence these perceptions. In addition, the development of targeted intervention strategies to improve student wellbeing is essential. We would like to emphasise that we have already started an exploratory study on this topic, which reinforces our commitment to progress in this area of research.

7 – Link Between Lifestyle and Emergency Service Utilization

Our analysis showed that unexpected health problems and worsening of existing conditions were the main factors associated with emergency care use. These findings highlight the need for future research to clarify the role of modifiable lifestyle factors in emergency care use. In particular, it is essential to investigate which emergency department visits could be prevented by behavioural change, to assess the influence of health literacy on the adoption of preventive behaviours, and to develop specific interventions to reduce overuse of these services among students. These aspects are key to guiding policies and practices that promote more efficient use of health resources.

8 – Expanding the Theoretical Framework with Alternative Perspectives

We appreciate the suggestion to engage with a broader range of authors beyond validation studies. We acknowledge the important contributions of Pelikan et al. (2022) and prior research preceding the M-POHL initiative, which have critically examined the conceptualization and measurement of health literacy.

To strengthen our theoretical foundation, we have:

- Included references to Pelikan et al. (2022) and their extensive work on HLS-EU-47 and HLS-Q12.
- Discussed alternative perspectives on health literacy assessment, emphasizing both supporting and critical viewpoints.

We sincerely appreciate your thoughtful and detailed feedback. Your comments have greatly helped to improve the clarity, methodological transparency and theoretical depth of our study. We have clarified key methodological choices, expanded the theoretical underpinnings, and suggested important directions for future research.

We believe that these revisions significantly strengthen the manuscript and look forward to your comments. We remain at your disposal. Thank you very much.

Best regards.

From the authors,

Jorge Rosário
